# A general dual-pathway network for EEG denoising

**DOI:** 10.3389/fnins.2023.1258024

**Published:** 2024-01-24

**Authors:** Wenjing Xiong, Lin Ma, Haifeng Li

**Affiliations:** Faculty of Computing, Harbin Institute of Technology, Harbin, China

**Keywords:** EEG denoising, dual-pathway structure, general network model, light weight autoencoder, blind source separation

## Abstract

**Introduction:**

Scalp electroencephalogram (EEG) analysis and interpretation are crucial for tracking and analyzing brain activity. The collected scalp EEG signals, however, are weak and frequently tainted with various sorts of artifacts. The models based on deep learning provide comparable performance with that of traditional techniques. However, current deep learning networks applied to scalp EEG noise reduction are large in scale and suffer from overfitting.

**Methods:**

Here, we propose a dual-pathway autoencoder modeling framework named DPAE for scalp EEG signal denoising and demonstrate the superiority of the model on multi-layer perceptron (MLP), convolutional neural network (CNN) and recurrent neural network (RNN), respectively. We validate the denoising performance on benchmark scalp EEG artifact datasets.

**Results:**

The experimental results show that our model architecture not only significantly reduces the computational effort but also outperforms existing deep learning denoising algorithms in root relative mean square error (RRMSE)metrics, both in the time and frequency domains.

**Discussion:**

The DPAE architecture does not require a priori knowledge of the noise distribution nor is it limited by the network layer structure, which is a general network model oriented toward blind source separation.

## 1 Introduction

Scalp electroencephalogram (EEG) is a time-varying non-linear and non-stationary physiological signal with weak amplitude and is highly susceptible to contamination by extraneous noise, resulting in various artifacts (Sanei and Chambers, 2013). The effective removal of artifacts in scalp EEG and the preservation of the pristine scalp EEG information are of great significance for brain science research and clinical applications (Wolpaw et al., 1991; Oberman et al., 2005; Cai et al., 2020). Artifacts can be classified into two categories: physiological artifacts and non-physiological artifacts (Urigüen and Garcia-Zapirain, 2015). Physiological artifacts are usually caused by the activity of body parts close to the head, such as the eyes (Croft and Barry, 2000), muscles (Muthukumaraswamy, 2013), and heart (Dirlich et al., 1997). In addition, the relative motion of the subject’s head and the electrode cap can produce physiological artifacts (Oliveira et al., 2016). Non-physiological artifacts are usually caused by environmental and equipment factors, such as whether the electrodes are in good contact with the scalp, whether the acquisition equipment is in good operation, and utility interference. Non-physiological artifacts can be reduced by acquiring in a shielded room, filtering, and subject self-suppression of the head and other body movements. In contrast, the physiological artifacts of electrooculogram (EOG) and electromyogram (EMG) are difficult to remove by simple filtering methods due to the irregularity of their movements and the frequency band overlap with the commonly used scalp EEG rhythm signals and need to be processed by suitable artifact removal algorithms.

Classical artifact removal methods include independent component analysis (ICA) (Jung et al., 2000; Ullsperger and Debener, 2010), wavelet transform (WT) (Hamaneh et al., 2014; Zhao et al., 2014), empirical mode decomposition (EMD) (Huang et al., 1998; Patel et al., 2016), and common average reference (CAR) (Ludwig et al., 2009). Among them, the hybrid optimization method is a newer research hotspot for scalp EEG denoising, which mainly focuses on EEG noise reduction by combining the classical algorithms mentioned above to extract different artifact component features. Mahajan and Morshed (2015) combined blind source separation and wavelet transform, which avoids distortion in reconstructing scalp EEG signals due to the removal of useful EEG information. Bono et al. (2014) proposed the WPT-EMD and WPT-ICA algorithms by combining the concepts of wavelet packet transform (WPT), EMD, and ICA, which has a significant increase in artifact removal ability using signal-to-noise ratio as a measure. Chen et al. (2014) combined the ensemble empirical mode decomposition (EEMD) and the joint blind source separation (JBSS) for muscular artifact cancelation in single-channel EEG recordings, which demonstrated the performance of these single-channel solutions. Islam et al. (2020) proposed an approach based on ICA to remove motion-related artifacts in the context of epilepsy and succeeded in improving the accuracy of epilepsy detection and prediction.

The advantages of applying the classical scalp EEG signal denoising methods mentioned above include high interpretability and good removal of specific artifacts, but the computational complexity is large and the generalization and robustness of the methods are not strong. The advantages of the generalization and robustness of deep learning are rapidly making it become a new development direction for scalp EEG noise reduction methods (Mumtaz et al., 2021). Yang et al. (2018) proposed a shallow multilayer perceptron (MLP) network for removing scalp EEG artifacts, which applies to the automatic removal of online scalp EEG by training the network offline, with the advantage of channel-independence and strong generalization ability. Anon Hanrahan (2019) used the one-dimensional convolutional neural network (CNN) to reduce noise in scalp EEG signals. Based on the signal-to-noise ratio (SNR) before and after denoising, this method has successfully surpassed principal component analysis (PCA). The complex CNN network structure of EEGdenoiseNet proposed by Zhang et al. (2020) outperformed the EMD algorithm in denoising the standardized EEG artifact dataset constructed by them. Sun et al. (2020) used a one-dimensional residual convolutional neural network (1D-ResCNN) model for denoising the raw time-domain waveforms of scalp EEG, which was effective in removing ocular artifacts but ineffective for myogenic artifacts. Zhang et al. (2021) proposed a new CNN network structure combining progressively increasing feature dimensions and downsampling for EMG denoising. The results show that structure design can help avoid overfitting and perform better.

In general, the removal of scalp EEG artifacts by deep learning is not controlled by the number of electrodes and does not require *a priori* knowledge of physiological artifact reference channels or some artifact signals, but due to the complexity and multiple sources of artifact signals, achieving good results for both ocular and myogenic artifacts using existing deep learning methods is difficult. These deep learning network models are complex and computationally intensive. In light of the recent explosion in portable BCI (brain-computer interfaces) (Guneysu and Akin, 2013; Nakanishi et al., 2017; Zhang et al., 2019; Wang et al., 2023), we suggested a lightweight scalp EEG noise reduction network model based on autoencoders. Autoencoders were first introduced by Hinton and Salakhutdinov (2006) as neural network trainers to reconstruct its input and are widely used in computer vision (Sinha et al., 2018), audio processing (Leglaive et al., 2020), and natural language processing (Lebret and Collobert, 2015). To alleviate the problem that the classic autoencoder tends to be overfitted, Vincent et al. (2010) proposed the denoising autoencoder. The denoising autoencoder uses data corrupted by noise to reconstruct the original data, which makes the abstract features more robust. Here, we modify the encoder module of the noise-reducing self-encoder. Instead of stacked encoding layers, we design parallel dual-pathway network layers with different neuron shrinkage ratios and combine them with a symmetry fusion block module. In this way, we construct a robust feature extraction encoder to remove the non-linear and multi-source scalp EEG artifact noise features and obtain a high-level representation of clean EEG. Key contributions of this work are summarized by:

- A general dual-pathway network structure separately was built using MLP, CNN, and RNN for automatic scalp EEG artifact removal and we demonstrate that it outperforms other deep learning methods.
- Modeling of scalp EEG contaminated with artifacts based on coupled systems.
- Blind source separation without *a priori* knowledge of the source signal.

The rest of the paper is organized as follows. Section 2 presents the scalp EEG artifact coupled system model, the dual-pathway autoencoder network separately constructed using MLP, CNN, and RNN, and the benchmark scalp EEG artifact dataset. Section 3 presents different evaluation metrics, whose results show that our approach outperforms other deep learning denoising methods. Also, we conducted ablation experiments for the proposed new network structure and validated the denoising performance on multichannel real EEG data. Concluding remarks follow in Section 4.

## 2 Methods and materials

### 2.1 The scalp EEG artifact hypothesis

As a time-varying non-linear and non-stationary signal, scalp EEG is susceptible to exogenous artifacts and physiological artifacts. Physiological artifacts are caused by a variety of internal body sources: EOG, EMG, electrocardiographic (ECG), etc. Traditional methods assume that the artifacts and scalp EEG signals are statistically independent of each other and that the signals we observe are composed of linear combinations of source signals as equation (1) (Parra et al., 2005), where f denotes the loss function of the transmission medium (skull) to the clean EEG signal and $g_{i}$ denotes the loss function of the transmission medium (skin surface) to each artifact signal.


(1) $$\text{Signal}=f\left(eeg\right)+\sum_{i=1}^{N}\mu_{i}*g_{i}\left(\text{artifac}t_{i}\right)$$

**Figure 1:**
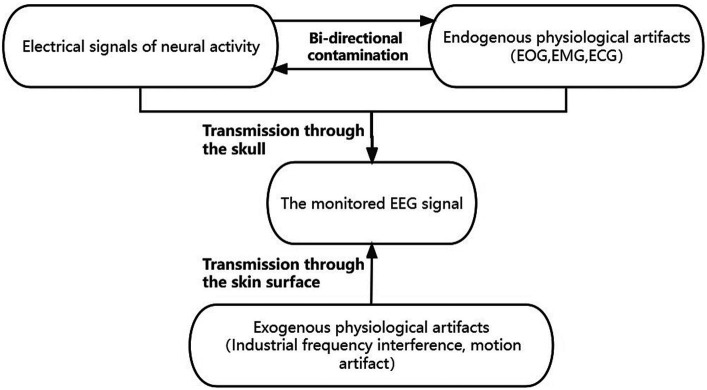
The coupled system model of scalp EEG contaminated by artifacts. The endogenous signals contain clean scalp EEG signals and physiological artifacts dominated by electrooculogram (EOG), electromyogram (EMG), and electrocardiographic (ECG). The endogenous signals antagonize each other and are co-transmitted through the skull to the scalp electrodes to be acquired. The exogenous signals, which contain physiological artifacts dominated by industrial frequency interference and motion artifacts, are transmitted via the scalp to the scalp electrodes, and together with the endogenous signals, constitute the observed scalp EEG. The exogenous signals comprised primarily of industrial frequency interference and motion artifacts are conveyed through the scalp to the scalp electrodes. These signals, in conjunction with the endogenous signals, collectively form the observable scalp EEG.

In reality, however, there is a bidirectional contamination between scalp EEG and artifacts, forming a coupled system (David and Friston, 2003). Exogenous artifacts can be removed by adjusting the acquisition environment and filtering. In contrast, endogenous artifacts and clean scalp EEG activity signals are interacting with each other during transmission through the scalp to the electrodes and are more difficult to remove. This can be modeled as equation (2):


(2) $$\text{Signal}=f\left(eeg+\sum_{i=1}^{M}\mu_{i}*g_{i}\left(\text{artifac}t_{i}^{\text{endo}}\right)\right)+\sum_{j=1}^{K}\delta_{j}*g_{j}\left(\text{artifac}t_{j}^{exo}\right)$$

where $\text{artifac}t_{i}^{\text{endo}}$ denotes endogenous artifacts and $\text{artifac}t_{j}^{exo}$ denotes exogenous artifacts. $g_{i}$ and $g_{j}$ represent the transmission loss functions for endogenous and exogenous artifacts of different transmission mediums, respectively. We wished to construct a network structure that can simulate this mutual antagonism of the source signal during the scalp transmission process. The schematic diagram illustrating the coupling model framework of scalp electroencephalography can be found in Figure 1. Therefore, we propose the following dual-pathway denoised autoencoder model. We fit the coupling between the two types of loss functions, by means of a multiscale dual-pathway structure and a fusion block to extract the feature coding of clean scalp EEG signals.

### 2.2 Network structure

Considering the coupled system, the clean scalp EEG signals and the artifact signals interfere and contaminate each other, which ultimately constitutes the observed scalp EEG signals composed of signals from multiple sources. In this paper, a dual-pathway autoencoder (DPAE) network architecture is designed for extracting the encoding of the target signal which is also the clean scalp EEG signal.

Firstly, at the time of extracting the primary features, the original input is mapped to subspaces of different dimensions to obtain feature representations at different scales. Secondly, through the fusion module, the joint representation features of different scales are compressed and reconstructed to obtain the scale-independent common feature information. Finally, the clean scalp EEG signal is reconstructed based on this common feature information. Notably, we associate the neural network layers at both ends of the fusion module through residual connections, i.e., the input and output layers of the fusion module are summed before feeding into the decoder. In view of the fact that the depth of the network structure increases with the addition of the fusion module, the residual connection can ensure that there is no gradient vanishing in the backpropagation (Drozdzal et al., 2016). Furthermore, the residual connection is added to construct an identity mapping of the joint representation based on the residual connection, which is more conducive to the model learning the common feature information in the different scale features. This model structure has the advantage of automatically modeling the contamination relationship between multiple interference source signals. It also learns multiscale feature representations in a dual pathway format and uses the coding of the target source signals as its learning target. This helps the fusion module to obtain common feature representations at different scales that contain more information about the target source signals.

**Figure 2:**
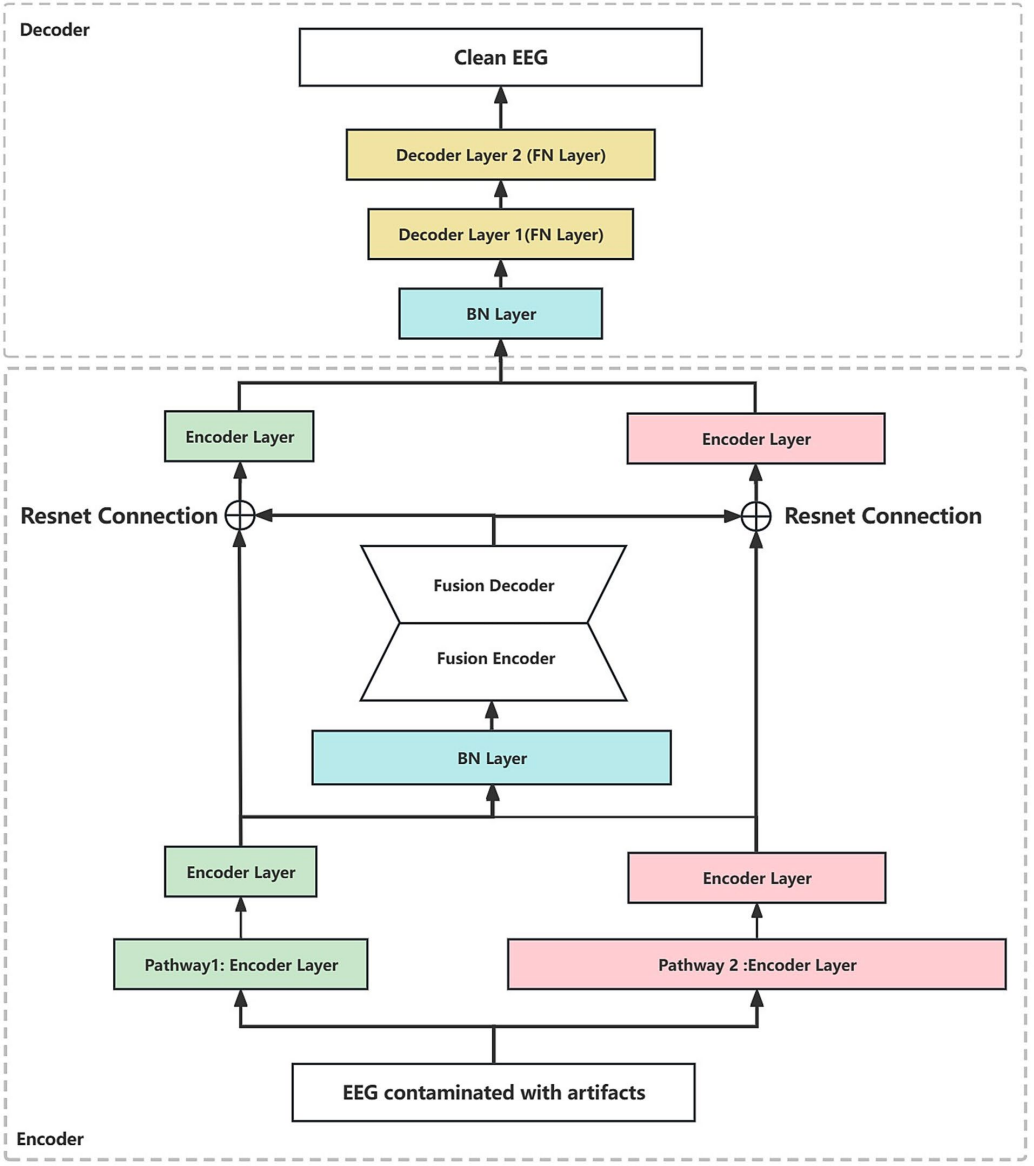
The dual-pathway denoising autoencoder (DPAE) structure diagram. The DPAE model first extracts multiscale abstract features by stacking network layers of varying scales. Subsequently, it simulates the coupling of multiple source signals through the fusion block. Following this, it employs residual connection structures to extract scale-independent generic encoding features of the target source signals. Finally, it reconstructs the target source signals based on the decoder. It is worth noting that to make the network structure look more intuitive, we ignore the joint representation layer. FN denotes a fully connected hidden neural layer, BN denotes a batch normalization layer, and the widths of the individual layers indicate the relative proportions of the layer neuron sizes. The encoder layers can be replaced by MLP, CNN, and RNN layers.

In addition, to better illustrate the generalization of the network frame, the dual-pathway autoencoder network architectures based on MLP, CNN, and RNN are constructed in this paper, respectively. Specifically, taking CNN as an example, we construct two sets of convolutional layer pathways with different sizes of convolutional kernels (1×3 vs. 1×5) and different steps (1×2 vs. 1×4) on the input layer of the network, respectively. In contrast to MLP, two sets of fully connected layer pathways are constructed with different neuron shrinkage ratios (0.45 vs. 0.75). Compared to RNN, two sets of GRU (gate recurrent unit) pathways are constructed with different unit shrinkage ratios (0.45 vs. 0.75). The two hidden layer pathways are followed by the fusion module. The fusion module is constructed based on a fully connected layer, which constructs the joint representation based on the two hidden layer pathways, and reconstructs the joint representation by compression coding first and then reconstructing the joint representation. The whole fusion module is a symmetric structure, as shown in Figure 2. The reconstructed joint representation is further compression coded by combining the residual connections after the fusion module. Finally, decoding is performed based on the decoder to synthesize clean scalp EEG signals. In addition, we added a batch normalization (Santurkar et al., 2018) layer after the joint representation layer every time, which is to regularize the features from two pathways to a uniform interval, reduce the degree of data divergence, and construct independently and identically distributed recombination features.

In terms of parameter details, if we take the number of neurons at the input as 512, the specific parameters of the DPAE network model based on MLP are shown in Table 1. Additionally, it should be noted that the number of neurons in the high-dimensional layer is 682 (512/0.75) and the number of neurons in the low-dimensional layer is 230 (512*0.45). This is to map the input data to the low and high dimensional spaces, respectively, and thus ensure feature variability extracted by the subsequent dual-pathway structure. After that, the shrinkage ratio of the two pathway network layers stacked is always set to 0.45 and 0.75, respectively, so the shape of each network layer can be calculated in turn. In other words, our pathway with the high ratio is the first to carry out an expansion operation to upgrade dimensionality and a few contraction operations to reduce dimensionality. This is intended to improve the features’ expressiveness. In contrast, in the low ratio pathway, it is all shrinkage operations. In the decoder, the two-path network layers are concatenated together, then one layer of the network layers is stacked and output directly.

**Table 1:** Parameters of each layer of the DPAE model.

Layer	Shape	Connected to
Input	512	-
Dense_layer1 (Path1)	230	Input
Dense_layer2 (Path1)	103	Dense_layer1
Dense_layer3 (Path1)	46	Dense_layer2
Dense_layer4 (Path2)	680	Input
Dense_layer5 (Path2)	511	Dense_layer4
Dense_layer6 (Path2)	383	Dense_layer5
Concatenate layer1	429	Dense_layer3, Dense_layer6
Batch normalization Layer1	429	Concatenate layer1
Dense_layer7 (fusion encoder)	193	Batch normalization Layer1
Dense_layer8 (fusion encoder)	86	Dense_layer7
Dense_layer9 (fusion feature)	39	Dense_layer8
Dense_layer10 (fusion decoder)	86	Dense_layer9
Dense_layer11 (fusion decoder)	193	Dense_layer10
Dense_layer12 (Path1)	46	Dense_layer11
Add layer1 (Path1)	46	Dense_layer3, Dense_layer12
Dense_layer13 (Path1)	20	Add layer1
Dense_layer14 (Path2)	383	Dense_layer11
Add layer2 (Path2)	383	Dense_layer6, Dense_layer14
Dense_layer15 (Path2)	287	Add layer2
Concatenate layer2	307	Dense_layer14, Dense_layer15
Batch normalization Layer2	307	Concatenate layer2
Dense_layer16 (decoder)	256	Batch normalization Layer2
Output	512	Dense_layer16

We propose a training strategy to facilitate the learning of the clean scalp EEG characteristic features from scalp EEG contaminated with artifacts. We designed clean scalp EEG and contaminated scalp EEG input-to-output pairs. We set clean EEG as the expected output and constructed contaminated scalp EEG frames with different signal-to-noise ratio EMG and EOG artifacts as the input. The dual-way autoencoder is trained in a supervised manner, using the Adam optimizer (Zhang, 2018) with the mean square error between the outputs and the expected reconstructed clean EEG as a loss function. Furthermore, all activation functions are SeLu.

### 2.3 Datasets and pre-processing

#### 2.3.1 Introduction to the dataset

**Figure 3:**
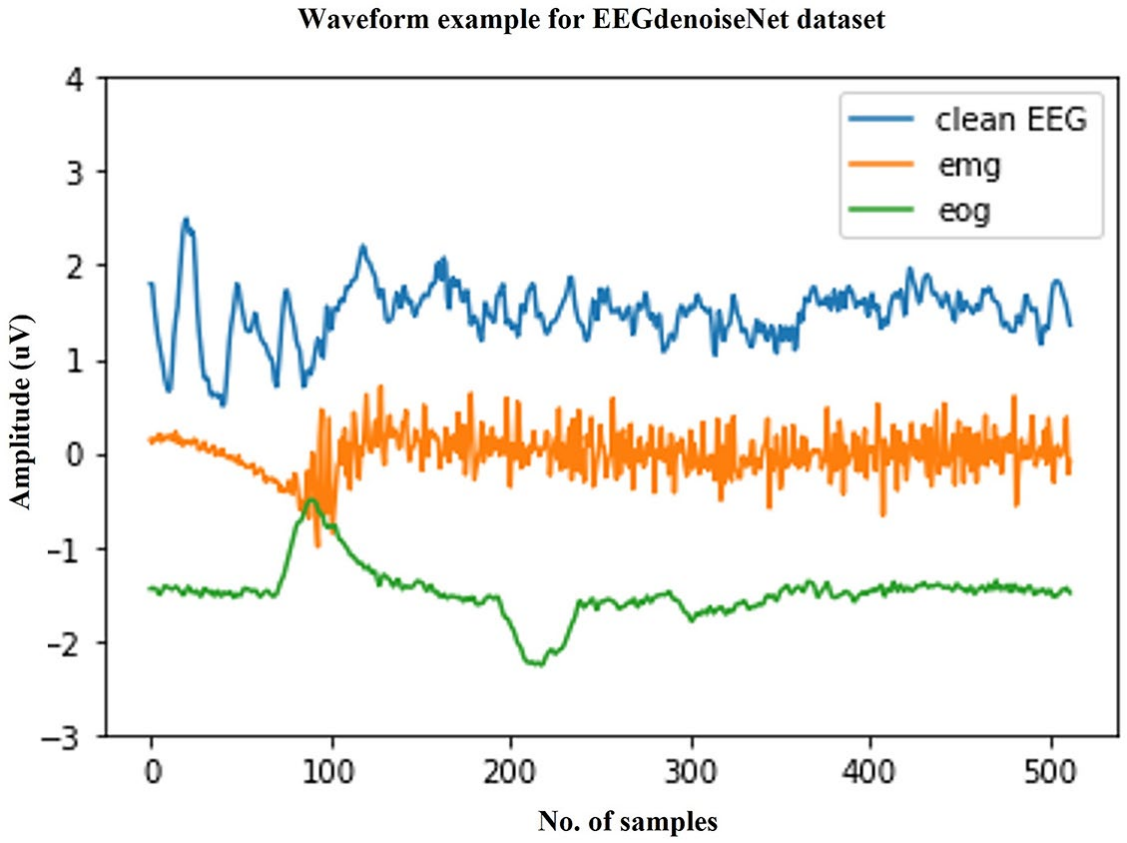
Typical examples in the EEGdenoiseNet dataset. The waveforms of clean scalp EEG, EOG, and EMG with a sampling rate of 256 Hz and a duration of 2 s are given here, respectively.

We selected a publicly available standardized scalp EEG artifact removal dataset to validate the effectiveness of the method. The EEGdenoiseNet is a benchmark dataset for scalp EEG artifact removal by deep learning. This dataset has been manually annotated to obtain single-channel pure EEG, EOG, and EMG, and has been standardized for segmentation. Figure 3 shows example waveforms of scalp EEG, EOG, and EMG, respectively. The total of 13,512 segments of scalp EEG with a sampling rate of 256 Hz consist of 4,514 segments of pure EEG, 3,400 segments of oculomotor artifacts, and 5,598 segments of EMG artifacts, respectively.

The clean scalp EEG signals in this dataset were not spontaneous. They were extracted from 52 subjects performing real and imagined motor tasks. The sampled data from the 64 electrode channels were first extracted from the clean scalp EEG signals by ICA and then segmented into 2 s one-dimensional EEG signals after band-pass filtering from 1 to 80 Hz. In other words, the final single-channel scalp EEG signal was extracted from different channels of EEG. The EOG signal was obtained from additional EOG channels from multiple publicly available datasets and processed by band-pass filtering from 0.3 to 10 Hz. The EMG signal was obtained from a publicly available facial EMG dataset and processed by band-pass filtering from 1 to 120 Hz.

With this dataset, we compared our method with the current state-of-the-art methods in power spectral density (PSD), signal-to-noise ratio (SNR), and other classical time series similarity measures for quantitative metrics.

#### 2.3.2 Pre-processing of the dataset

As mentioned previously, the EEGdenoiseNet dataset is divided into clean EEG, EOG, and EMG datasets, all with a sampling rate of 256 Hz, and all have been segmented in advance for a 2 s time window. We randomly selected 80% of the clean EEG as the training set and the remaining 20% of the EEG as the test set. The EEG contaminated by EOG and EMG is linearly constructed according to equation (3), which is also the noise-added formula used in the original dataset paper. Additionally, it should be noted that the sample counts of EEG, EMG, and EOG were not equal, we adopted a random sampling strategy without replacement for EMG and EOG to construct EEG contaminated with artifacts.


(3) $$EEG_{\text{Contaminated}}=EEG_{\text{clean}}+\lambda *\text{Artifac}t_{EMG/EOG}$$

where λ is the artifact coefficient inferred from the SNR of the noise-added EEG. The SNR value is calculated as equation (4), with lg representing the logarithm with a base of 10.


(4) $$SNR=10\mathrm{lg}\left(\frac{RMS\left(EEG_{\text{clean}}\right)}{RMS\left(\lambda *\text{Artifac}t_{EMG/EOG}\right)}\right)$$

RMS denotes the root mean square and represents the power of this segment of the signal, calculated as equation (5).


(5) $$RMS\left(\text{Signal}\right)=\sqrt{\frac{1}{N}\sum_{i=0}^{N}\text{Signa}l_{i}^{2}}$$

Therefore, the smaller the value of λ, the larger the SNR value. The authors who proposed this dataset set the signal-to-noise ratio interval to [-7 dB, 2 dB], and we adopted the same setting scheme. As a side note, the data segmentation of our EMG-contaminated EEG is as follows: set the number of training sets for each SNR level to 4,478 and the number of test sets to 560. Set the data segmentation of our EOG-contaminated EEG as follows: set the number of training sets for each SNR level to 2,720 and the number of test sets to 340. This is also consistent with the way the authors constructed the segmentation of this dataset. We also set up separate validation sets equal to the size of the test set.

## 3 Results

### 3.1 Performance evaluation metrics

Here, we adopted the evaluation criteria established by the creators of the dataset, consistent with their criteria for assessing denoising performance. Evaluating the network performance in terms of the root mean square error (RRMSE) of correlation on the time and frequency domains of the denoised EEG data, as well as the Pearson correlation coefficient values with the paired clean EEG.

First, we calculate the RRMSE of the denoised EEG from both the time domain and frequency domain, respectively. Here, we denote the denoised EEG by $f\left(y\right)$ and the paired clean EEG by x as ground truth. Then, the RRMSE in the time domain is calculated as equation (6):


(6) $$\text{RRMS}E_{\text{temporal}}=\frac{RMS\left(f\left(y\right)-x\right)}{RMS\left(x\right)}$$

Considering that the scalp EEG signal is a non-linear time-variant signal, the RRMSE in the frequency domain introduces the power spectral density for calculating as equation (7):


(7) $$\text{RRMS}E_{\text{spectral}}=\frac{RMS\left(PSD\left(f\left(y\right)\right)-PSD\left(x\right)\right)}{RMS\left(PSD\left(x\right)\right)}$$

The Pearson correlation coefficient (CC) is the classic measure of correlation of time-series data and is calculated as equation (8):


(8) $$CC=\frac{cov\left(f\left(y\right),x\right)}{\sqrt{Var\left(f\left(y\right)\right)Var\left(x\right)}}$$

**Figure 4:**
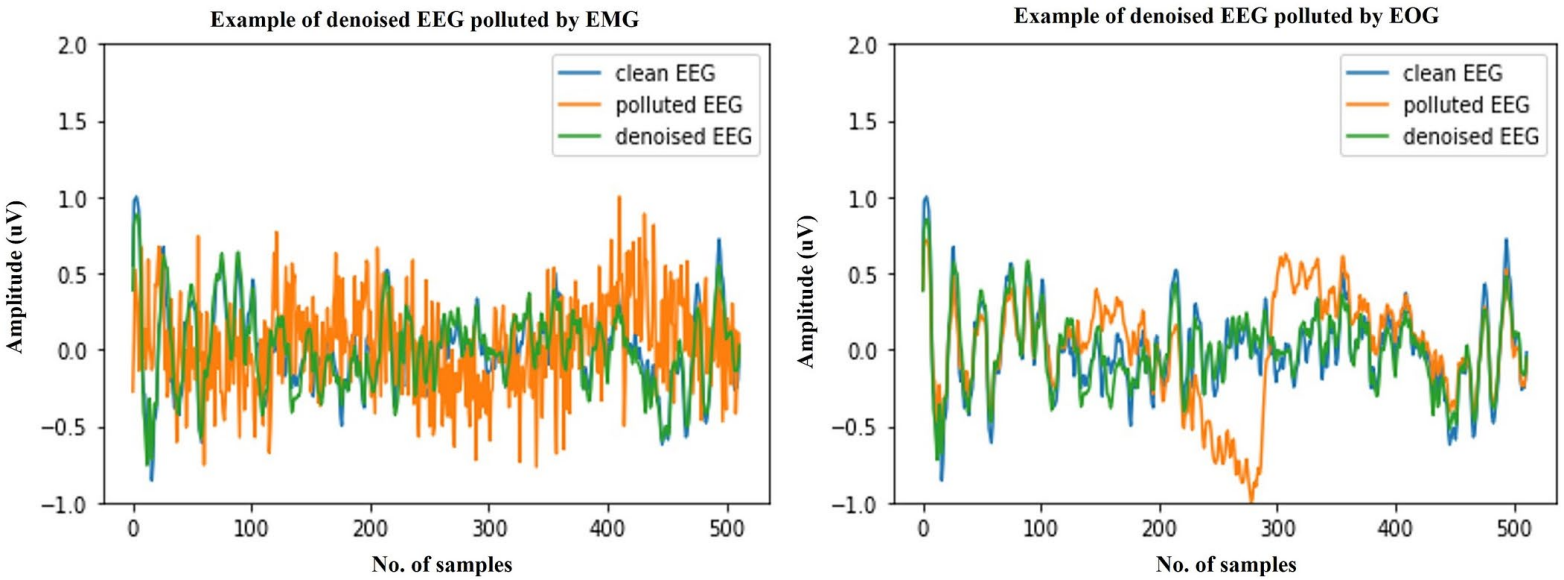
Example waveforms of the denoising results of EMG (left) and EOG (right). It is evident that the time-domain waveforms of the denoised EEG signal closely resemble those of the clean scalp EEG signal, irrespective of the presence of contamination from EOG or EMG.

**Figure 5:**
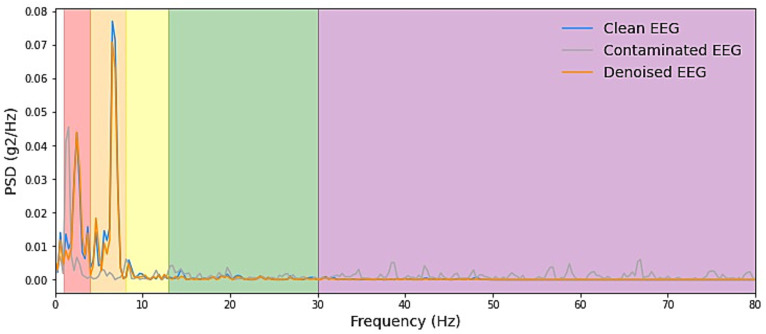
Example PSD plots of denoising result of EMG. We presented power spectral density (PSD) plots for scalp EEG signals contaminated by EMG across various frequency bands both before and after noise reduction. The delineated frequency intervals for each band are as follows: delta [1–4 Hz], theta [4–8 Hz], alpha [8–13 Hz], beta [13–30 Hz], and gamma [30–80 Hz]. The efficacy in eliminating artifacts in the higher-energy EEG bands, specifically delta, theta, and alpha, exhibited greater strength and displayed more consistent curves. Conversely, in the beta and gamma bands, the ability to remove artifacts was less pronounced due to the comparatively lower energy levels inherent in the clean EEG signals within these bands.

**Figure 6:**
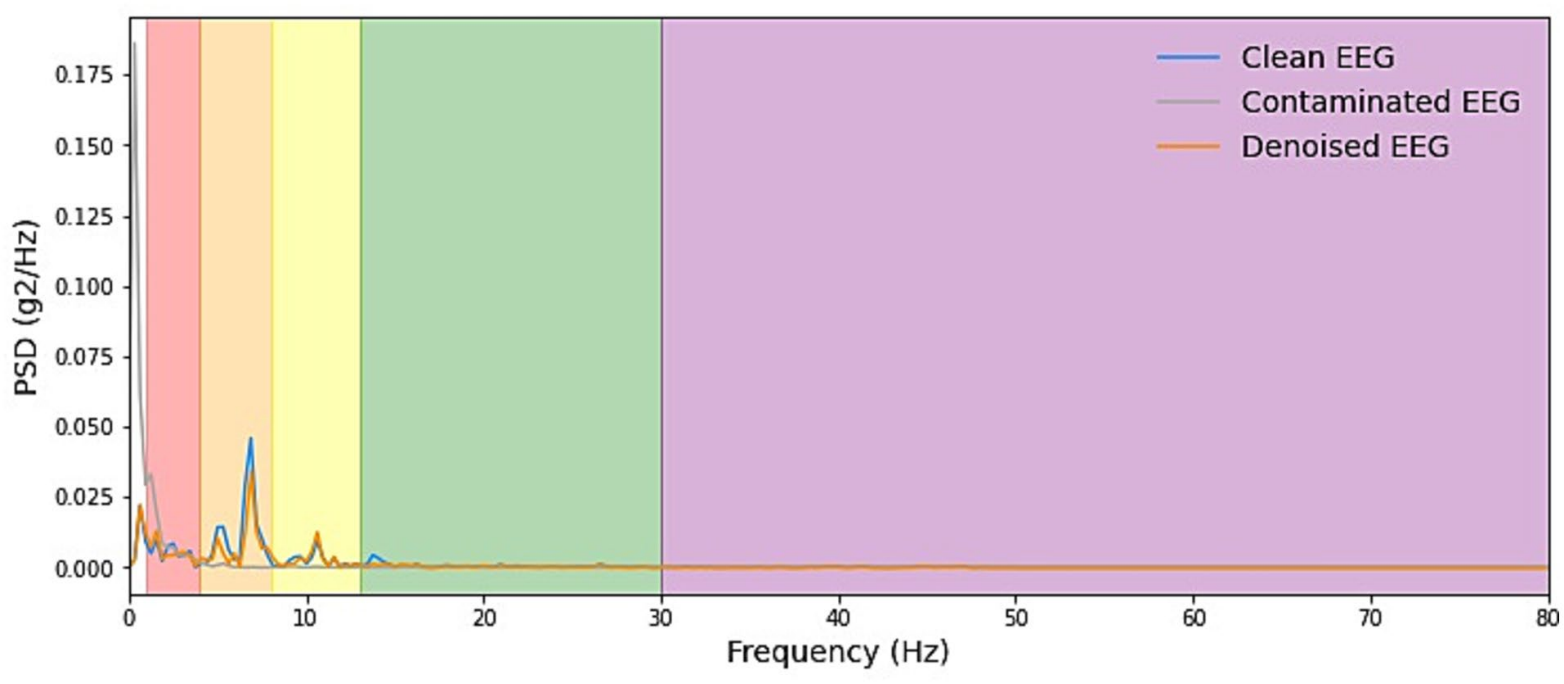
Example PSD plots of the denoising results of EOG. We present power spectral density (PSD) plots for scalp EEG signals contaminated by EOG across various frequency bands both before and after noise reduction. The delineated frequency intervals for each band are as follows: delta [1–4 Hz], theta [4–8 Hz], alpha [8–13 Hz], beta [13–30 Hz], and gamma [30–80 Hz]. The frequency range of EOG typically falls within 0–13 Hz, which is relatively narrow. As depicted in the figure, EOG primarily introduces interference in the delta, theta, and alpha frequency bands. The results demonstrate that our denoising method consistently exhibits exceptional performance across all frequency bands.

We start by explaining the source of the test data, which was constructed with the training set by sampling without replacement, which ensures that for the testing stage, either clean scalp EEG, EOG, or EMG are completely new and unseen. Some samples of our model noise reduction performance are given in Figures 4–6. For each segment of data, the normalization method has two steps: subtracting the standard deviation and then dividing by the maximum of the absolute values. It is important to highlight that when presenting the results of noise reduction through power spectrograms, we differentiated the various frequency bands of the EEG by employing distinct colors. This was done to accurately depict our method’s capacity to preserve the essential information within the power spectrum of each band. The frequency distribution intervals of each band are as follows: delta [1–4 Hz], theta [4–8 Hz], alpha [8–13 Hz], beta [13–30 Hz], and gamma [30–80 Hz].

### 3.2 Contrast with other deep learning methods

To have a more comprehensive view of the performance of our model, we compare it with a fully convolutional neural network (FCNN), simple CNN, complex CNN (Emara et al., 2019), and RNN, which are proposed with the benchmark dataset and the latest novel CNN. Table 2 shows the scales in parameters and FLOPs (floating point operations) (Frølich and Dowding, 2018) of each network. Firstly, considering the number of parameters, it is evident that our methods belong to the forefront, characterized by a minimal parameter count. However, compared with the RNN model, the number of parameters is slightly increased, considering that the RNN only has three hidden layers, while our DPAE model has 16 hidden layers, so it can be said that the DPAE framework can increase the depth of the network while effectively reducing the number of parameters. FLOPs represent the amount of network computation when the model is propagated forward, which can be used to measure the computational complexity of the model. It can be seen that the FLOPs of the three network frameworks of the DPAE are extremely small, and are one-tenth or even one-twentieth of the other models, among which the FLOPs value of the DPAE MLP model is the smallest. This indicates that our method consumes very little memory and is very fast, which is conducive for applications.

**Table 2:** Comparison of the network scales.

Model	FCNN	RNN	Simple CNN	Complex CNN	Novel CNN	DPAE MLP	DPAE 1D-CNN	DPAE 1D-RNN
Params	1.0 M	**0.7 M**	16.8 M	8.5 M	33.6 M	1.5 M	2.0 M	2.3 M
FLOPs	57.4 M	55.3 M	53.7 M	20.1 M	124.5 M	**3.1 M**	3.9 M	5.8 M

We highlights the minimum values for parameters and FLOPs through bold formatting.

**Figure 7:**
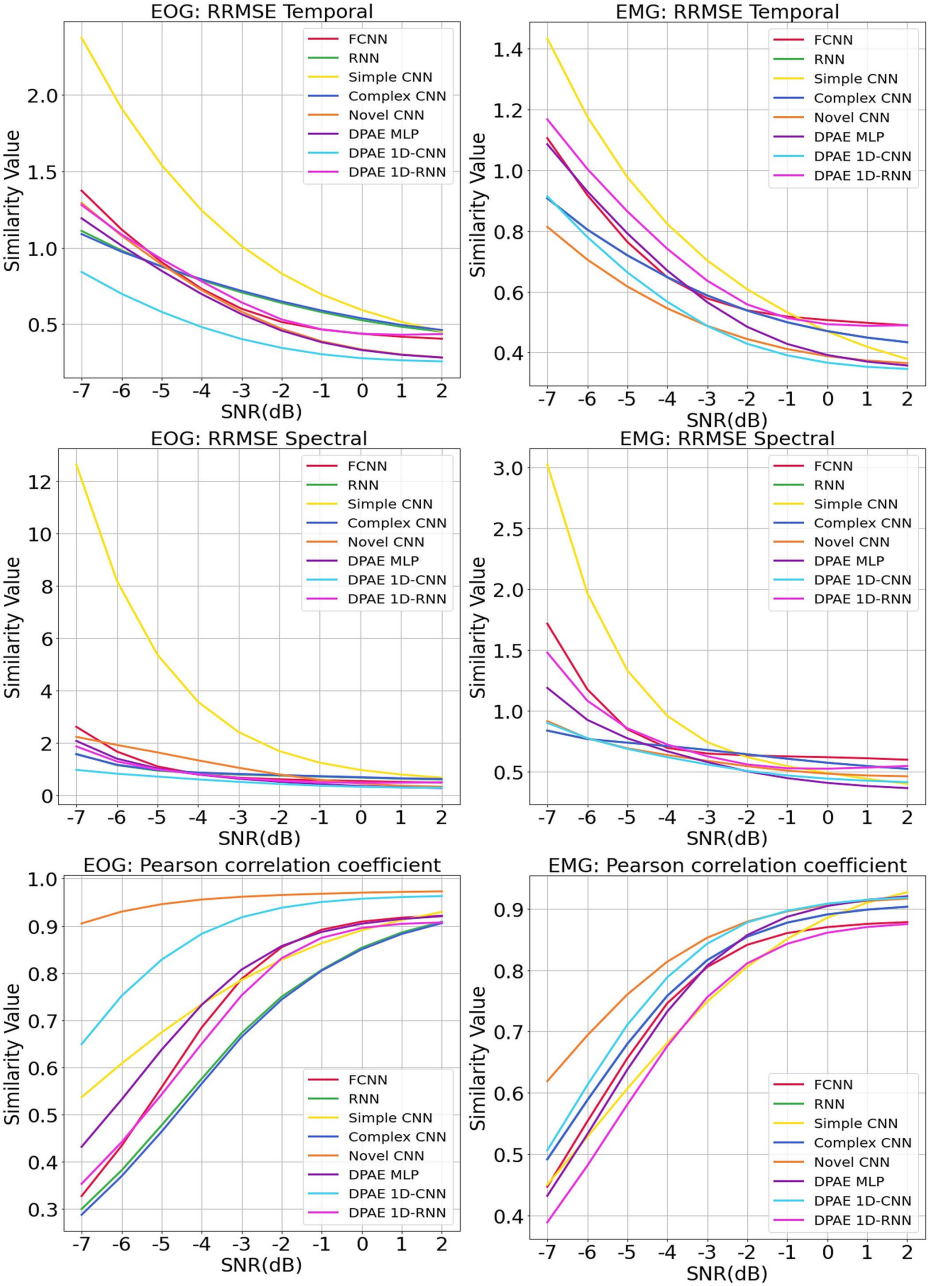
Evaluation metrics curves of the denoising results from different artifacts of the EEGdenoiseNet dataset. The figure presents the denoising results of different neural network models based on time-domain and frequency-domain root relative mean square error (RRMSE) and Pearson correlation coefficients. These results are obtained for scalp EEG signals contaminated by varying signal-to-noise ratios of EOG and EMG.

We trained the above methods all in 200 epochs with batch size at 128, using the same optimizer and loss function as our method. We present the benchmark assessment metrics proposed with this dataset against the RRMSE temporal, RRMSE spectral, and Pearson correlation coefficients graphs at all SNR levels in Figure 7. The first column gives the performance for scalp EEG contaminated by EOG under the three noise reduction metrics, while the second column corresponds to the noise reduction results for EEG contaminated by EMG.

In terms of trend, we can see that our noise reduction performance gets better as the SNR level increases. The fluctuation of our method is much more stable compared to other methods, which indicates the higher robustness of our method. The DPAE CNN model stably outperforms other models at all SNR levels in both temporal and spectral RRMSE metrics for EMG removal. The DPAE CNN also obtained the best performance on the removal of EOG as the signal-to-noise ratio went beyond -3 dB. In addition, the DPAE MLP model and the DPAE RNN model significantly outperform the baseline FCNN and RNN models, confirming the generalization of the DPAE frame across network structures. Secondly, the DPAE MLP model stably outperforms other deep learning algorithms on EOG removal after the SNR level exceeds -6 dB both on RRMSE temporal and spectral. In EMG removal, it outperforms other models in RRMSE spectral after the SNR level exceeds -3 dB, and in RRMSE temporal after the SNR level exceeds zero. The DPAE RNN, on the other hand, has a weaker performance improvement and does not outperform other deep learning algorithms, but only manages to completely outperform the simple CNN and outperform the complex RNN at the same SNR levels. As a side note, our method does not perform well on the Pearson correlation coefficient.

### 3.3 Ablation study

**Figure 8:**
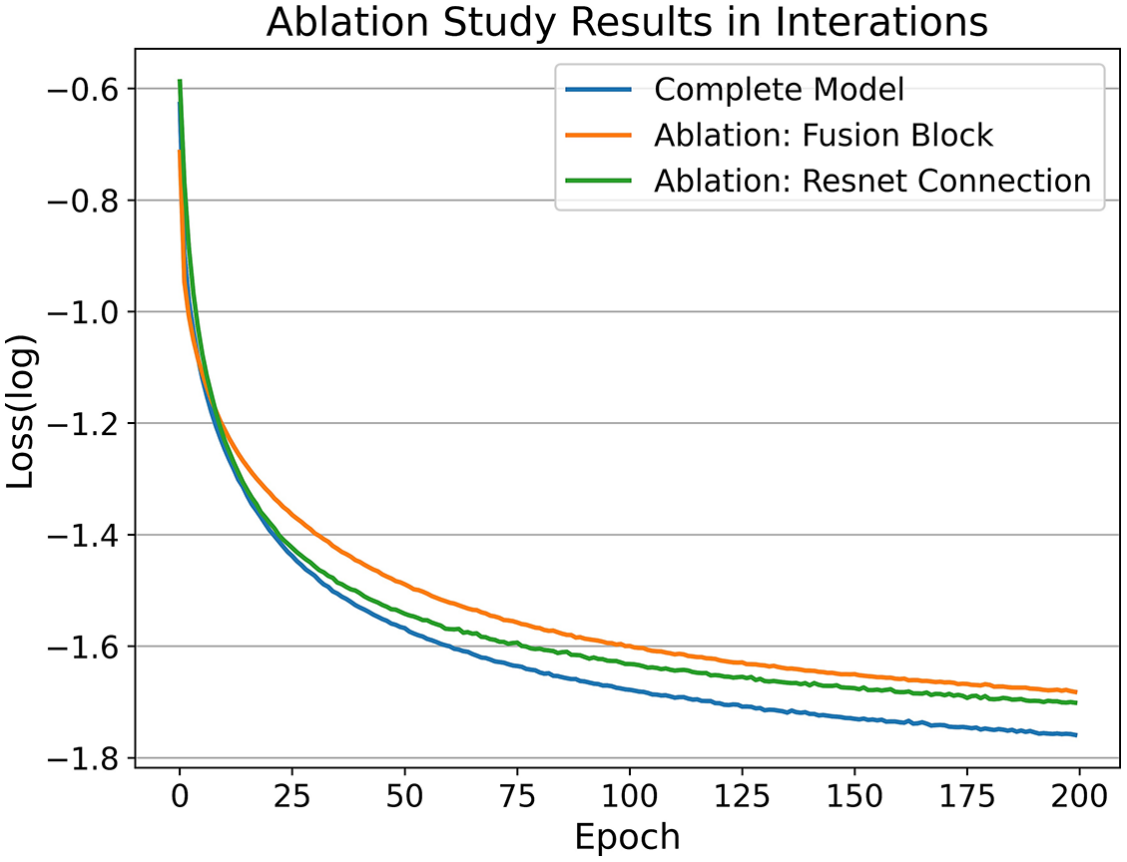
Ablation study on loss comparison. We conducted the ablation study on two separate instances: one involved the removal of the fusion block module, and the other involved the removal of the residual connection. Both instances utilized the same random seed and the same training set (utilizing the EMG dataset). The observed metric is the training losses.

In addition, our encoder design scheme mainly consists of three elements: a dual-pathway network structure, fusion block module, and residual connection. We took the two-pathway network as the baseline and the complete network as the ablation object based on the MLP layer. The ablation study (Meyes et al., 2019) was set on two occasions: removing the fusion block module or removing the residual connection. In terms of control variables, we set the three networks under the same random seed with the same training set (using the EMG dataset) and consistent hyperparameters (batch size = 128, leaning rate = 0.001, epoch = 200). The experimental results are shown in Figure 8.

As we can see, the model with the fusion block removed has the slowest convergence speed and the worst final loss level. The model with the residual connections removed has a slightly higher convergence speed, and the final loss is better but not as good as the complete model. First, this result is consistent with the fact that residual connections can improve the convergence speed of the network. Second, it shows that the fusion block module can indeed effectively fuse and update the coding representation of scalp EEG signals, which can not only improve the convergence efficiency of the network jointly with the residual connection but also make the network converge to a better local optimum. Finally, according to the results of the comparison between eliminating the fusion block and eliminating the residual connections for the network performance degradation, the fusion block contributes more to the performance improvement of the neural network compared to the residual connections.

### 3.4 Multichannel noise reduction experiment

The clean scalp EEG of the EEGdenoiseNet dataset is one-dimensional although it comes from different electrode channels. That is, our model is currently not based on specific channels for noise reduction, but in the form of single-channel reuse of the same model. However, most of the real scalp EEG datasets are multichannel, coupled with the fact that the clean scalp EEG signals of EEGdenoiseNet extracted by ICA differ from the real scalp EEG data. Therefore, in order to verify the practicality of our method, we did another experiment on multichannel scalp EEG noise reduction.

The scalp EEG data were obtained from the BCI Competition IV dataset 2a (Brunner et al., 2008), a four-class motor image dataset with nine subjects and 288 trials per subject. The number of electrodes was 22, and the sampling rate was 250 Hz. We randomly sampled 192 trials of data from two subjects (with equal proportions for the four motor imagery tasks). We intercepted 2 s of EEG data from which the subjects started motor imagery and resampled the scalp EEG to 256 Hz as ground truth. Those data were then combined with EMG data from EEGdenoiseNet to simulate contaminated EEG data at different signal-to-noise levels.

**Figure 9:**
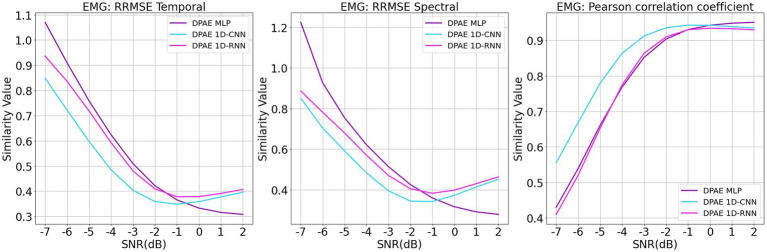
Denoising results of myoelectric artifacts on multichannel scalp electroencephalogram at different signal-to-noise ratios. The figure presents the denoising results of different DPAE models based on time-domain and frequency-domain root relative mean square error (RRMSE) and Pearson correlation coefficients. These results are obtained for multichannel scalp EEG signals contaminated by varying signal-to-noise ratios of EMG.

For the training of the network model, we made the following changes: the original 1d DPAE model structure was still used, and the data from each trial were fed into the network channel by channel for noise reduction. Figure 9 shows the noise reduction results of the three model frameworks under different signal-to-noise levels. It can be seen that all three models maintain consistent noise reduction with the EEGDenoseNet dataset at different signal-to-noise ratio levels when facing real multichannel EEG data. Among them, 1dCNN DPAE consistently outperforms the other two models in EMG artifact removal until the signal-to-noise level is below 0 dB. This further illustrates the greater robustness of the 1dCNN DPAE model. The MLP DPAE model, on the other hand, maintains the trend of increasing noise reduction ability with increasing signal-to-noise ratio, whereas the other two models show a decrease in noise reduction ability at signal-to-noise ratios greater than 0 dB, suggesting that the MLP DPAE model is more capable of generalization.

## 4 Conclusion

In this paper, we propose a general dual-pathway autoencoder network for automatic scalp EEG denoising and validating the model separately constructed using MLP, CNN, and RNN on benchmark EEG datasets.

The experimental results show that the DPAE model constructed based on the above three architectures significantly outperforms the model constructed on the basic architecture, proving the effectiveness of our method. Secondly, the DPAE CNN model outperforms the existing deep learning noise reduction methods in terms of RRMSE temporal and spectral at almost all SNR levels, both in the EOG removal experiments and in the EMG removal experiments. Both the DPAE MLP and DPAE RNN also show competitive performance. Finally, in the comparison of the number of parameters in the network model and the computational complexity, the scale of our model is much smaller than existing deep learning methods, which proves the efficiency of the DPAE framework. Our method has the advantage of not limiting the number of channels and performs stably on the noise reduction of 22-channel EEG data. However, we have not yet started noise reduction experiments on real-time data, which is one of our future enhancement directions. In this way, we can combine the advantages of a lightweight network and apply it to portable brain–computer interface devices.

Our feature extraction structure of dual pathways, combined with residual connection and constant mapping of fusion modules, enables the DPAE model to efficiently guide the network to learn scale-independent common feature representations of the target source signals for reconstruction. Our network model does not require *a priori* distributional knowledge of multiple source signals and can be well migrated for application to blind source signal separation.

Also, since the overall framework of our method is an autoencoder, it is not difficult to envision that the encoder structure and weight design in this paper can be used as an upstream task for deep learning of scalp EEG data based on transfer learning without complicated manual filtering and can be applied directly to remove artifacts to improve the performance of downstream tasks.

## Data availability statement

The original contributions presented in the study are included in the article/supplementary material, further inquiries can be directed to the corresponding author.

## Ethics statement

The studies involving humans were approved by the Research Ethics Committee of Keio University. The studies were conducted in accordance with the local legislation and institutional requirements. The participants provided their written informed consent to participate in this study.

## Author contributions

WX: Conceptualization, Data curation, Formal analysis, Investigation, Methodology, Software, Validation, Visualization, Writing – original draft, Writing – review & editing. LM: Resources, Supervision, Writing – original draft, Writing – review & editing. HL: Conceptualization, Funding acquisition, Methodology, Resources, Supervision, Writing – review & editing.
