# Supplementary material for: A general dual-pathway network for EEG denoising
Source: Front Neurosci. 2024 Jan 24;17:1258024. doi: 10.3389/fnins.2023.1258024 (PMC10847297; doi:10.3389/fnins.2023.1258024)
Supplement: Supplementary file 1 [file Data_Sheet_1.docx]

Supplementary Material

**Code Availability**

The data supporting the findings of this study were obtained from the publicly available resources detailed below:

1.EEGdenoiseNet Repository:

Repository URL: https://github.com/ncclabsustech/EEGdenoiseNet

2.Code for DPAE Repository:

Repository URL: https://github.com/doubleX612/Code-for-DPAE

For transparency and accessibility, the code used in this study is openly accessible through the provided GitHub links.
